# Derivatization of PVA into Polyols Suitable for Fabrication of Rigid Polyurethane Foams—Preliminary Studies and Perspectives

**DOI:** 10.3390/ma18122780

**Published:** 2025-06-12

**Authors:** Jacek Lubczak

**Affiliations:** Department of Organic Chemistry, Faculty of Chemistry, Rzeszów University of Technology, Al. Powstańców Warszawy 6, 35-959 Rzeszów, Poland; jml@prz.edu.pl

**Keywords:** poly(vinyl alcohol), glycidol, ethylene carbonate, polyol, polyurethane foams, properties, biodegradation

## Abstract

Polyols derived from poly(vinyl alcohol) (PVA) have not been reported before. The hydroxyalkylation of PVA with oxiranes leads to powdered or gum-like products that are not miscible with isocyanates and therefore useless as sources of polyurethane foams. Glycidol and ethylene carbonates were used to dissolve and convert PVA into liquid polyol. The physical properties of the PVA-derived polyol, such as the density, viscosity, and surface tension, were determined. The polyol was then used to obtain rigid polyurethane foams (PUFs). Foaming conditions were optimized, and the apparent density, volume water uptake, dimensional stability, heat conductance coefficient, pore size, thermal resistance, compressive strength, and glass transition temperature of the obtained PUFs were determined. The properties of the obtained PUFs were similar to those of classic rigid PUFs, but the thermal resistance of the former is better. Specifically, PVA-derived PUFs are thermally resistant at temperatures of up to 150 °C. Furthermore, they are ecologically safe; in standard soil conditions, 54.6% or 100% biodegradation of the foams in cube and powder form, respectively, was observed, as measured by BOD after 28 days of storage.

## 1. Introduction

Poly(vinyl alcohol) (PVA) finds applications as a component in adhesives, textile finishes [1], and varnishes, and as a stabilizer for emulsion paints [2]. It is also used as a component in eye drops and artificial tears and for the production of dental floss [3,4,5], protective gloves, foils and coatings [6], plates, and pipes resistant to hydrocarbons and oils. PVA is a non-toxic compound and biodegrades slowly. It is prepared by the hydrolysis of poly (vinyl acetate); therefore, some percentage of the unconverted ester meres can be expected to be present in commercial PVA [7]. There are various PVAs available, containing 98% (efficient hydrolysis product), 86–92% (medium hydrolysis product), and 70–80% vinyl alcohol units.

The hydroxyl groups of PVA seem to enable the use of this polymer to obtain PUFs, provided that the PVA can be converted into a liquid resin with the hydroxyl groups still available. Such resins have not been reported before. However, the hydroxyalkylation of PVA with ethylene and propylene oxides (EO and PO) and glycerol epichlorohydrin had already been described by the 1950s [8]. It has been found that products containing 25 weight % of the attached ethylene oxide are powders that are visually not different from PVA, while those containing between 25 and 50% are gum-like polymers. Above 35%, the derivatives are semi-transparent, light-amber gums. All derivatives are highly soluble in water.

Attempts to graft ethylene oxide on PVA were made in a high-pressure reactor at 80 °C for 12, 17, and 30 h, with a two-fold weight excess of EO related to PVA powder. It was also noticed that the PVA + EO reaction at an initial temperature of 100 °C resulted in an exothermic effect, causing the temperature to jump to 200 °C [9] and leading to the isolation of a thick, dark-brown tar product. When the PVA + EO reaction was induced with ethyl acetate for 6.6 h at 90 °C, a cream powder was isolated, containing 10% of attached EO [9]. The reaction at 92–97 °C for 6 h with the continuous addition of EO did not yield liquid products; instead, a light-yellow powder was obtained [10]. A PVA + EO reaction was also conducted in water, and an elastic and well-plasticized product was obtained [11]. Later, it was found that ethoxylated PVA reacted with 2,4-toluilene diisocyanate and water to produce a spongy solid product that absorbed water well [12].

Glycerol epichlorohydrin was applied as a crosslinking agent for PVA ethers [13] and also for PVA epoxidation to obtain adhesive binders [14]. The reaction of PVA with EO and glycerol epichlorohydrin in water in the presence of sodium hydroxide and sulfuric (VI) acid catalysts was described in [15]. The obtained polymers had good water solubility and a low glass transition temperature. They were used to obtain elastic foils with good tensile strength.

Alkylene carbonates such as ethylene carbonate (EC) and propylene carbonate (PC) are well-known hydroxyalkylating agents [16]. However, they have not been used thus far for the hydroxyalkylation of PVA. In [17], it was shown that PO reacted as a crosslinking agent for the copolymer ethylene–vinyl alcohol when added at the 5% level [17]. Up to now, PVA hydroxyalkylation products with alkylene carbonates suitable for the synthesis of polyurethane foams have not been described. In this work, we tested them with various hydroxyalkylating agents to successfully isolate derivatized PVA. A liquid polyol was obtained, its structure and properties were investigated, and it was used to obtain rigid PUFs.

## 2. Experimental Section

### 2.1. Materials

The following materials were used in this work: PVA (87% hydrolyzed, average molecular weight *Mv*~30,000–70,000 Da; Sigma-Aldrich, Taufkirchen, Germany); glycidol (GL, pure 98%; Sigma-Aldrich, Taufkirchen, Germany); ethylene carbonate (EC, pure, ≥99%; Fluka, Buchs, Switzerland); potassium carbonate (anal. grade 100%; POCH, Gliwice, Poland); polymeric diphenylmethane 4,4′-diisocyanate (pMDI; Merck, Darmstadt, Germany); triethylamine (TEA, anal. grade ≥ 99%; Fluka, Buchs, Switzerland); and surfactant Silicon L-6900 (pure; Momentive, Wilton, CT, USA).

### 2.2. Synthesis of Polyols

A mixture of substrates (4.4 g PVA, 37.0 g GL, and 97 g EC) was heated at 130 °C with mechanical stirring (2000 rpm). After the dissolution of the PVA, the mixture was heated to 180 °C. The reaction was followed by the determination of the epoxide number (EN) in order to determine whether all the GL was consumed. After that, 1.0 g of K_2_CO_3_ catalyst was added at 80 °C. A further reaction (with EC) was performed at 180 °C until almost all the EC was consumed, down to 1% of its initial value.

### 2.3. Analytical Methods

The reaction of PVA with GL was monitored via epoxide number determination using hydrochloric acid in dioxane [18]. The progress of the hydroxyalkylation reaction with EC was monitored using the barium hydroxide method described in [19]. Finally, the hydroxyl number (HN) of polyol was determined by acylation with acetate anhydride in dimethylformamide [20]. The ^1^H-NMR spectra of the reagents were recorded at 500 MHz with a Bruker UltraShield instrument (Rheinstetten, Germany) in DMSO-d_6_ and D_2_O with hexamethyldisiloxane as the internal standard. The IR spectra were registered on an ALPHA FT-IR BRUKER spectrometer (Ettlingen, Germany) in KBr pellets or by the ATR technique. The MALDI–ToF spectra of polyols were obtained on a Voyager-Elite Perceptive Biosystems mass spectrometer (Framingham, MA, USA) working in linear mode with delayed ion extraction, equipped with a nitrogen laser working at 352 nm. The method of laser desorption from gold nanoparticles (AuNPET LDI MS) was applied [21].

### 2.4. Physical Properties of Polyols

The density, viscosity, and surface tension of polyols were determined with a pycnometer, a Höppler viscometer (typ BHZ, prod. Prüfgeratewerk, Medingen, Germany), and the detaching ring method, respectively.

### 2.5. Obtaining the Polyurethane Foams

The foams were obtained from 10 g of polyol, 0.29 g surfactant (Silicon L-6900), 0.2–0.01 g catalyst (TEA), and water (2–3%). The mixture was homogenized, and 10.0–17.5 mg pMDI (containing 30% of tri-functional isocyanates) was added with vigorous stirring. After creaming ceased, the obtained foams were stored at ambient temperature for 3 days. Samples for physical studies were cut from the foams.

### 2.6. Properties of Foams

The apparent density [22], water absorption [23], dimensional stability at 150 °C temperature [24], thermal conductivity coefficient (IZOMET 2104, company IZOTOP, Nova Dubnica, Slovakia), and compressive strength [25] of the PUFs were measured. The thermal conductivity coefficient was measured at 20 °C after 72 h of PUF conditioning. Thermal analyses of foams were performed using the dynamic method in a ceramic crucible at a temperature range of 20–600 °C, with about 100 mg of sample, under an air atmosphere, using a Thermobalance TGA/DSC 1 derivatograph, Mettler, with a 10 °C/min heating rate. Topological pictures of PUFs were analyzed with a Panthera microscope (prod. Motic, Wetzlar, Germany).

### 2.7. Biodegradation of Foams

The biodegradation of the PUFs was tested with an OxiTop Control S6 instrument (WTW-Xylem, Rye Brook, NY, USA). The respirometric method was used to measure the oxygen demand necessary for the aerobic biodegradation of the polymeric materials in soil. The consumed oxygen was assessed using the value of biochemical oxygen demand (BOD), which is the number of milligrams of captured oxygen per mass unit of tested polyurethane material. Biodegradation tests were performed according to the norm [26]. For the biodegradation test, sieved and dried gardening soil was used with the following parameters: 5% humidity (according to ISO 11274-2019 [27]), pH = 6 (according to ISO 10390-2005 [28]), and particle diameters < 2 nm. *BOD* was determined for every sample, taking into account the BOD of the tested system reduced by the *BOD* of the soil and the concentration of the tested compound in the soil using the following formula:

(1) $$BODs=\frac{{BOD}_{x}-{BOD}_{g}}{c}$$

where *S* is the number of measurements (in days), *BOD_S_* is the biochemical oxygen demand of the analyzed sample within S days (mg/dm^3^), *BODx* is the biochemical oxygen demand of the measuring system (bottle with sample and soil) (mg/dm^3^), *BODg* is the biochemical oxygen demand of soil without a sample (mg/dm^3^), and *c* is the sample concentration in the tested system (mg/dm^3^). The degree of biodegradation of the polyol or the foam based on it was determined using the following formula:

(2) $$D_{t}=\frac{{BOD}_{S}}{TOD}\cdot 100\%\;$$

where Dt is the biodegradation degree of the sample (%) and *TOD* is the theoretical oxygen demand (mg/dm^3^).

For a compound of known C, H, N, and O percentage and the total mass of the sample, the TOD value can be calculated from the following equation:

(3) $$TOD=\frac{16\cdot [2C+0.5\cdot \left(H-3N\right)-O]}{m}\;$$

where *C*, *H*, *N*, and *O* are the mass fractions of the elements in the biodegraded material and m is the sample mass of the material (g).

## 3. Results and Discussion

### 3.1. Synthesis and Properties of Polyol

The key obstacle in the hydroxyalkylation of PVA is the dissolution of PVA in the reaction mixture and obtaining a liquid product. As was mentioned above, the products of PVA hydroxyalkylation with oxiranes were powders or gums. On the other hand, the reaction of PVA with oxiranes such as EO and/or PO requires an elevated temperature. The bps of EO and PO are 14 and 35 °C, respectively, which implicates using high-pressure equipment in the PVA + oxirane reaction. Using GL as a solvent (bp 167 °C) removed this obstacle. The hydroxyalkylation of PVA with a large excess of EC in relation to the PVA unit (1:15 or more) results in the formation of polyols suitable for obtaining elastic PUFs due to the long oxyalkylene chains which are grafted on the hydroxyl groups of the PVA unit [29]. On the other hand, using a lower excess of EC per PVA mere in order to obtain polyols suitable for the synthesis of rigid PUFs leads to the gelatinization of the semi-products, which is not suitable for their further fabrication into PUFs. The use of glycidol alone for the hydroxyalkylation of PVA causes the PVA to dissolve, but the obtained product is a semisolid resin, probably due to the hydrogen bond network, because every addition of GL to the PVA increases the number of hydroxyl groups by 1.

**Scheme 1:**
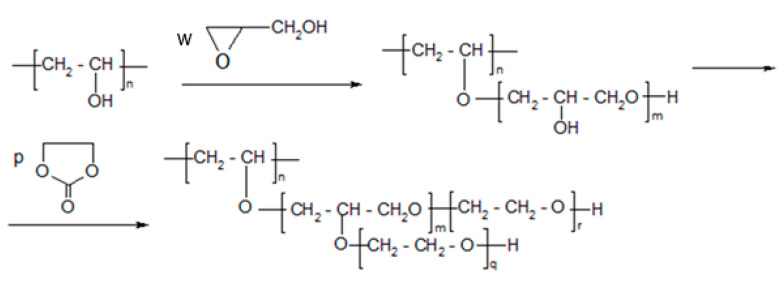
Polyol synthesis from PVA and two-step process, where w = n m and p = n m q + n r.

Thus, the advantage of using GL and EC together is that the semi-products of the PVA + GL reaction dissolve successively in EO and react with EO to elongate the oxyalkylene chain, and, at the same time, they decrease the “concentration of OH groups”, making the product liquid (Scheme 1).

**Figure 1:**
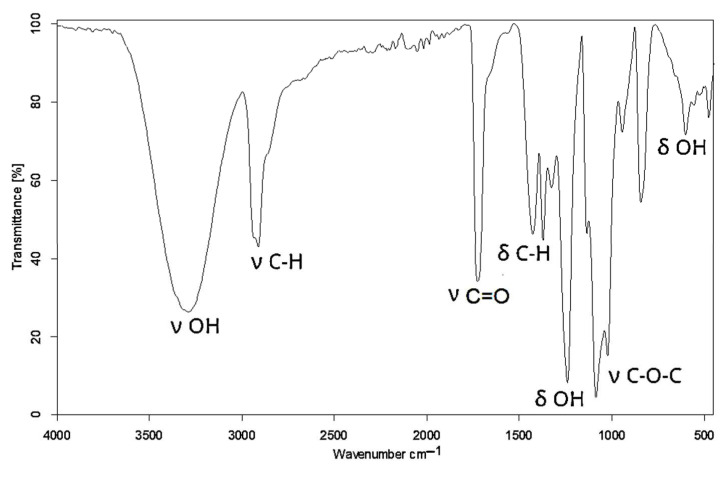
IR spectrum of PVA.

The reaction progress was monitored by IR, ^1^H NMR, and MALDI–ToF spectral techniques. In the IR spectrum of PVA, a broad band was observed at 3289 cm^−1^ from the hydroxyl group stretching vibration, as well as deformation bands at 1427 and 601 cm^−1^ (Figure 1).

**Figure 2:**
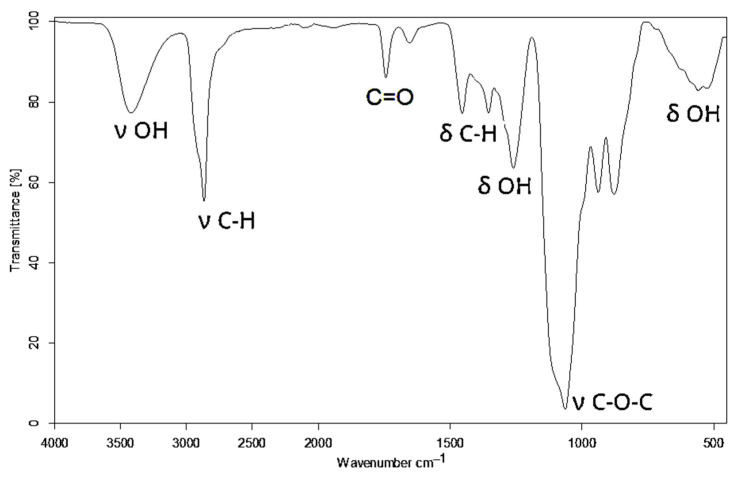
IR spectrum of polyol.

Additionally, the band corresponding to C-O in the hydroxyl groups was centered at 1086 cm^−1^. The remnant CH_3_COO- in non-hydrolyzed meres of PVA unfolded at 1725 cm^−1^ and 1240 cm^−1^ from the ester group C(O)-O. The formation of new C-O-C bonds upon the reaction between the hydroxyl groups of PVA and GL appeared in the IR spectrum of polyol at 1063 cm^−1^ (Figure 2). This confirmed the incorporation of new structural fragments formed by the ring opening of GL and EC upon the hydroxyalkylation of PVA.

**Figure 3:**
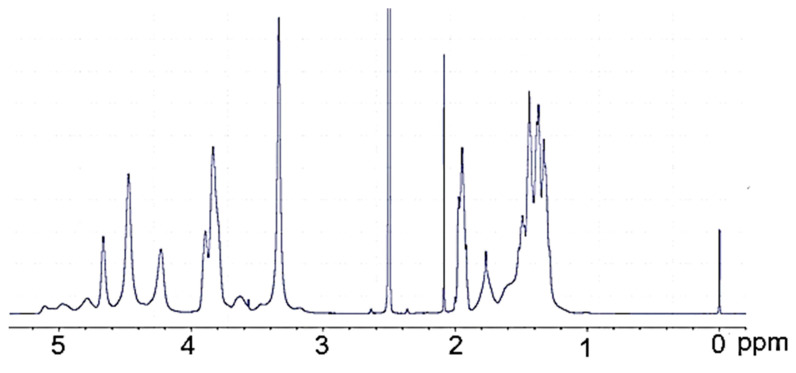
^1^H-NMR spectrum of PVA.

**Figure 4:**
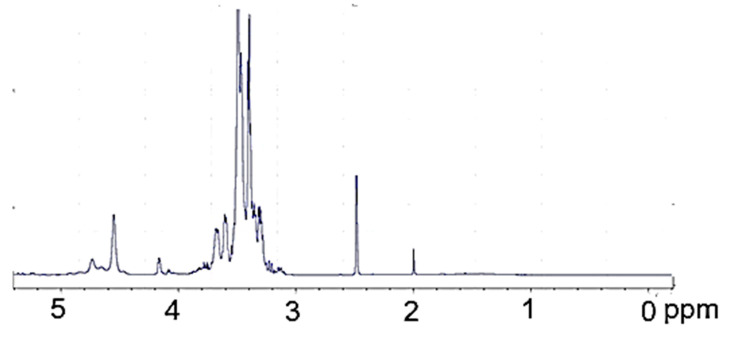
^1^H-NMR spectrum of polyol.

The ^1^H-NMR PVA (Figure 3) is surprisingly rich; the hydroxyl proton resonances are present at 4.22, 4.45, 4.64, and further within 4.7 to 5.1 ppm. The origin of these resonances was confirmed by their disappearance upon the addition of D_2_O. The methine resonance at 3.90 ppm comes from the hydrogen attached to the hydroxylated carbon. Methylene resonances present in the PVA chain give signals within 1.3–1.8 ppm. The methyl group of a remnant acetate group was present at 1.95 ppm. In the ^1^H-NMR spectrum of polyol (Figure 4), practically no methylene resonances within 1.3–1.7 ppm attributed to PVA were observed. Instead, a considerable increase in methylene resonances at 3.0–3.6 ppm was found due to GL and EC ring opening events during the hydroxyalkylation of PVA. It was also noted that hydroxyl group resonances within 4.22–5.10 ppm, previously observed in the spectrum of PVA, disappeared, while new hydroxyl proton resonances within 4.5–4.8 rose, attributed to hydroxyl groups derived from GL and EC upon ring opening. These signals were also confirmed by selective deuteration with D_2_O.

**Scheme 2:**
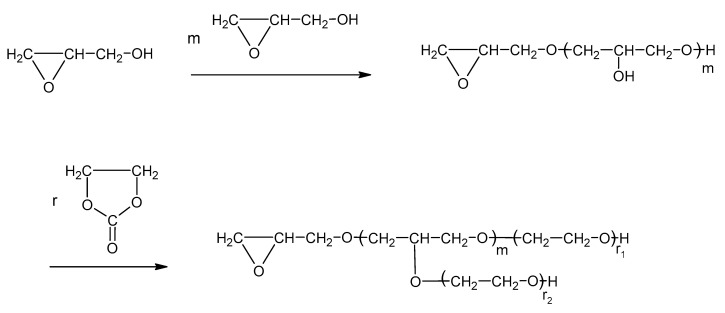
Oligomerization of GL and hydroxyalkylation of formed oligomers with EC, where r = m r_2_ + r_1_.

Low-molecular-weight products in the obtained polyol were identified using MALDI–ToF spectrometry (Table 1). It was found that in polyol, products of hydroxyalkylation of oligomers of GL with EC and the elimination of CO_2_ were present according to Scheme 2 (Table 1, entries 4, 5, 8, 10, 11,13, 14, 17, 19, and 20–25).

**Scheme 3:**
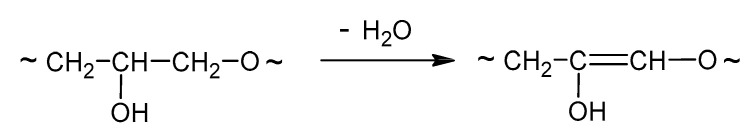
Dehydration of the product.

This low-molecular-weight product can undergo dehydration in the conditions of the reaction (Table 1, entries 12, 15, and 27; Scheme 3).

**Scheme 4:**
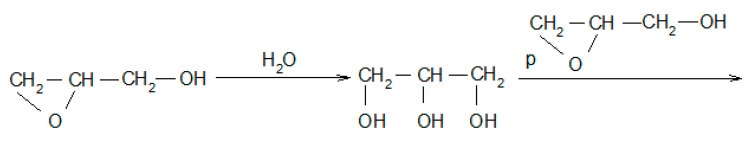

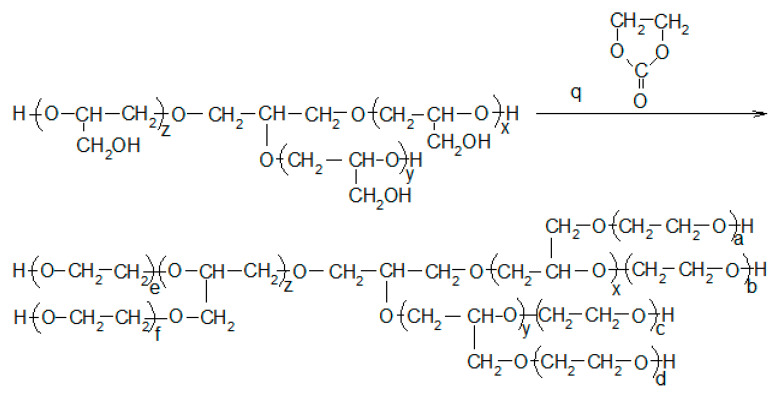
Water hydroxyalkylation reaction, where p = x + y + z and q = ax + b + dy + c + fz + e.

Moreover, the products of the oligomerization of GL itself can be identified (Table 1, entries 18 and 26; Scheme 2). Water can react with GL to give glycerol and further the consecutive products by reaction with GL and/or with EC (Table 1, entries: 2, 3, 6, 7, and 9; Scheme 4):

The obtained polyol was a light-brown slowly flowing resin. The following polyol properties were determined at 20 °C: density 1.300 g/cm^3^, viscosity 3090 mPa^.^s, and surface tension 38.5 mN/m. These parameters are within the typical region for polyols suitable to obtain PUFs [30]. The hydroxyl number of the polyol was 472 mg KOH/g, which suggested that the obtained polyol could be used to obtain rigid PUFs.

### 3.2. Polyurethane Foam

PUFs were obtained from polyol by reaction with pMDI and water in the presence of TEA as a catalyst and Silicon L-6900 as a surfactant. CO_2_ gas, obtained by reacting isocyanate with water, was used for foaming. The foaming mixture was optimized, and the final composition was adjusted to produce a rigid foam with small, regular pores (Table 2). It was found that foams with the best properties are obtained by using such an amount of pMDI that the molar ratio of isocyanate groups to hydroxyl groups (–NCO/–OH, isocyanate index) in the initial reaction mixture is 1.1 during foaming. The coefficient was determined based upon the hydroxyl number. When less pMDI was used, the obtained PUFs were under-crosslinked, with a viscous surface and low foaming. On the other hand, when more pMDI was used, the PUFs had irregular pores, often with an elongation towards the growing direction. The optimized amount of surfactant was 2.9 g/100 g polyol. A lower amount gave PUFs with irregular pores or even stopped the growth and caused the collapse of the PUFs. The optimized water content was 2–3% related to the mass of the polyol. A higher amount of water led to fragile PUFs with large pores. The best amount of catalyst was 0.3–0.4 g/100 g polyol depending on the amount of water applied in the foaming composition. This is related to the high reactivity of polyol containing primary hydroxyl groups, which are formed in the polyol during functionalization with EC. The increase in catalysts above 2% (related to the mass of the polyol) did not cause the pores’ elongation in the growing direction, but shortened the creaming and growing time of the PUFs, which is undesirable in the technological process. The optimal foaming composition contained 2.9 g surfactant, 2–3% water, and 0.3–0.4 g TEA as catalyst, all related to polyol mass with an isocyanate coefficient fixed at 1.1. In such cases, the cream time was 30 s, while growing times were 15 and 28 s for compositions with 2 and 3% water/100 g polyol, respectively. The drying time was very short: 1–2 s.

**Figure 5:**
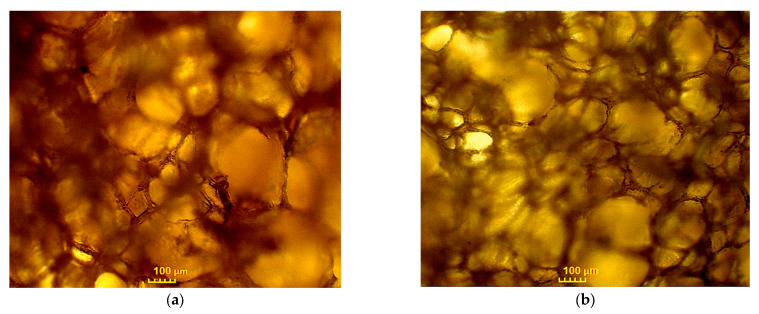
Optical microscopy images of foams with 2% (**a**) and 3% (**b**) water content/100 g of polyol.

The optimized PUFs were characterized in terms of apparent density, volume water uptake, dimensional stability, heat conductance coefficient thermal resistance, compressive strength, and glass transition temperature. The PUFs obtained with 2% water contribution had an apparent density of 76.0 kg/m^3^. The application of more water (3%) resulted in more carbon dioxide release and consequently a lower apparent density (56.1 kg/m^3^). The apparent density of the obtained PUFs places them in the category of rigid PUFs (Table 3). Water uptake after 24 h exposure was merely 2%, which suggests that such material can be used as an isolator in conditions of high humidity. The low water absorption indicates the presence of mainly closed pores in the foams. The obtained PUFs had good dimensional stability. When the PUFs were kept at 150 °C for 40 h, linear shrinkage was maximum at 3.52% (Table 4). Foams obtained with 2% water had an oval shape (Table 5 and Figure 5) with a larger diameter of 290 µm and a shorter one of 228 µm. Typical rigid PUFs usually have a pore diameter within 200–600 μm [31]. When 3 g of water was applied to the foaming composition, the pores were more spherical, with diameters of 197 and 169 µm. The thickness of the cells was 18 and 9 µm in PUFs obtained with 2 and 3% water in the foaming mixture, respectively. The heat conductance coefficient fell within 0.0328–0.0388 W/m·K, which reaches the upper limit of rigid PUFs (Table 3).

**Figure 6:**
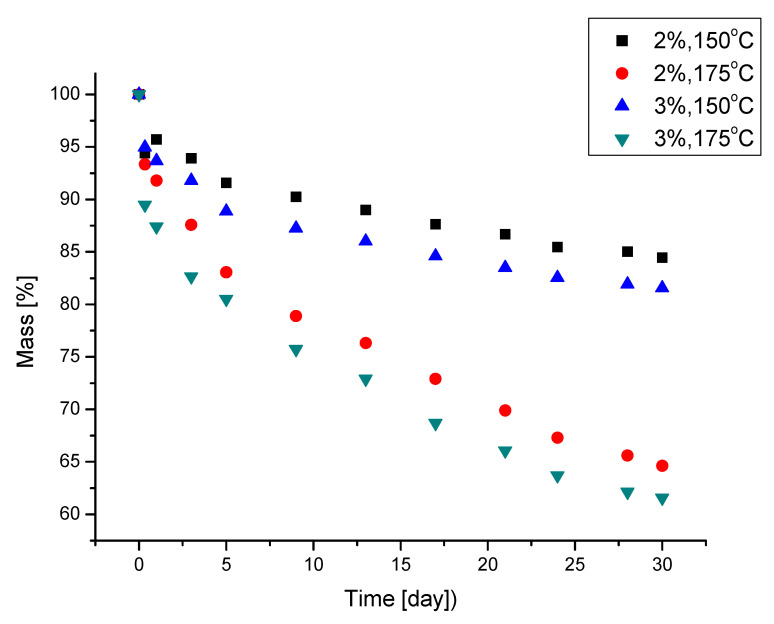
Thermal stability of polyurethane foams at temperatures of 150 °C and 175 °C (water content of 2% or 3% of the foaming compositions based on 100 g of polyol.)

The thermal resistance of the PUFs was determined by using mass loss after one month of exposure at 150 and 175 °C with the concomitant measurement of compressive strength after the exposure (Table 3). The compressive strength of the PUFs depended on their apparent density and was within 0.361 and 0.259 MPa, respectively, for the higher and lower apparent density of the PUFs. The wall thickness of the PUFs also influenced their compressive strength; it was lower in the case of PUFs obtained from 3% water in the foaming mixture. The compressive strength of the obtained PUFs was characteristic of rigid PUFs. During heating, the PUFs lost mass, especially within the first day of the test (Figure 6). One month of exposure led to a mass loss of 15.8 and 18.6% or 36 and 39% for PUFs obtained with 2% and 3% water, respectively. It was noticed that PUFs annealed for 30 days at 150 °C considerably increased in compressive strength by 20–27% (Table 3). This could be caused by the additional crosslinking of the PUFs. This was confirmed by the disappearance of isocyanate bands in the IR spectra of PUFs annealed at a temperature of 150 °C, which was also observed in our previous studies [32,33,34,35,36]. At 175 °C, an increase in the compressive strength of the PUFs also took place, but vast mass loss indicated a process of structural degradation of the PUFs. That ultimately resulted in the graphitization of the material and the formation of a structure of higher compressive strength. As we established in our previous systematic studies [32,33,34,35,36], the long-term heating of PUFs above 150° leads to their slow degradation, which consequently diminishes their mechanical resistance and graphitization, accompanied by the appearance of C=C bonds (observed in the IR spectra). Due to the graphitization of the PUFs, structural fragments are formed which are responsible for the increased compression strength of the annealed PUFs [37]. Depending on which of the mentioned processes prevails, an increase or decrease in the compression strength of the annealed PUFs can be found. In this paper, we observed an increase in the compression strength of the annealed PUFs with their concomitant degradation as identified by mass loss.

**Figure 7:**
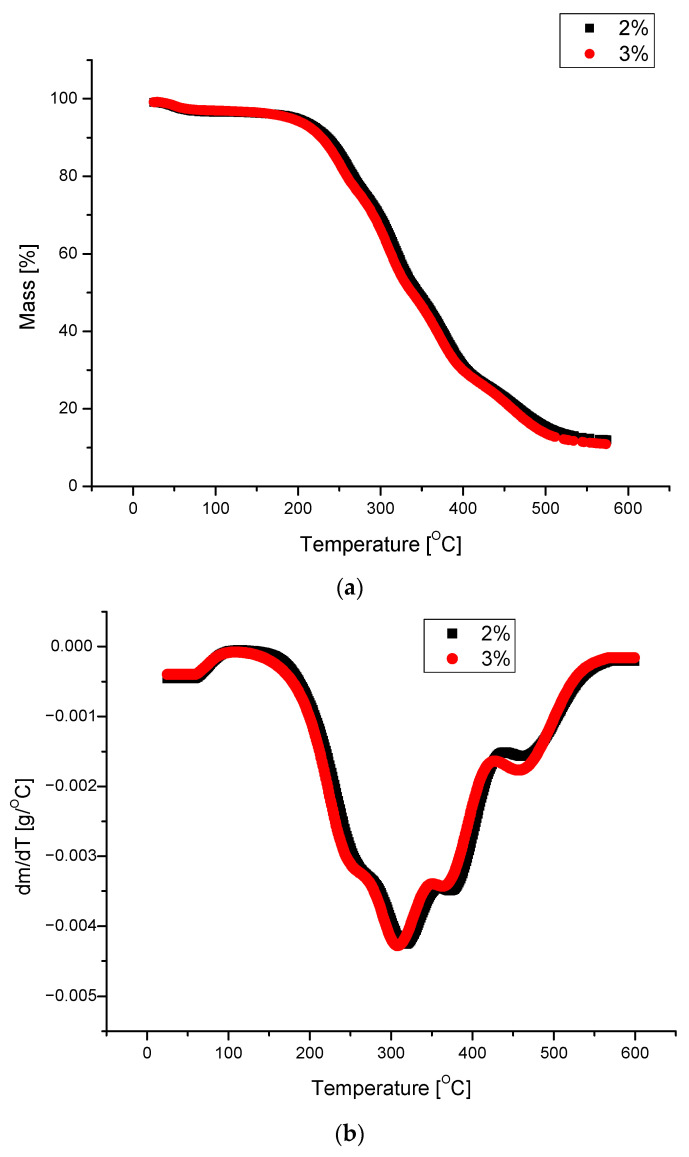
Thermal analysis of foams obtained from polyols: mass change as a function of temperature (**a**); differential mass change as a function of temperature (**b**). (Water content was 2% or 3% of the foaming compositions based on 100 g of polyol).

**Figure 8:**
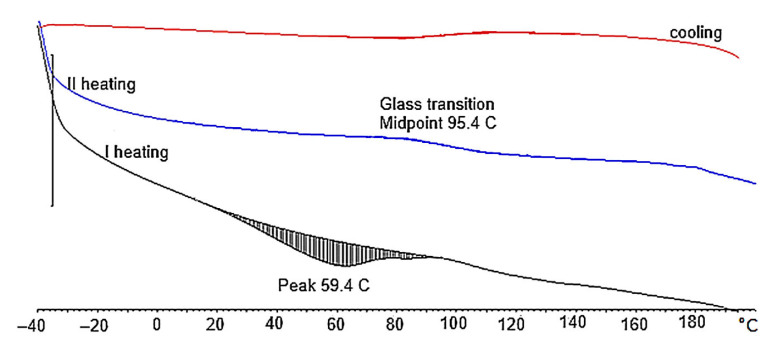
DSC thermogram of foam obtained from composition containing 2% water/100 g polyol.

Generally, the obtained PUFs can be used as working materials at 150 °C for a long time in comparison with typical rigid PUFs, which exhibit 21% mass loss after one day of exposure at 150 °C [38]. A dynamic thermogravimetric analysis demonstrated that the obtained PUFs lost 5% mass already at 190–197 °C, while a maximal decomposition temperature was observed within 310–320 °C (Table 6), which corresponds to the thermal decomposition of polyurethane into amine and carbon dioxide. The solid remnant after thermal exposure was 16% of the initial mass of the sample (Figure 7a). Three maxima were observed on the dm/dT vs. T graph (Figure 7b) at 300–310, 380, and 460 °C, with an additional shoulder at 260 °C. The latter corresponds to the thermal dissociation of urethane bonds. The decomposition of polyurethane to amine and CO_2_ is responsible for the first maximum [31], and the second maximum is attributed to the decomposition of the ether bond in the polyol, while the third maximum is due to the dissociation of the bond of the methylene aromatic ring (from pMDI) [39,40]. The DSC of the obtained PUFs provided further evidence of the physicochemical processes at lower temperatures. The endothermic peak at 15–100 °C (Figure 8) was attributed to the TEA and trace water (absorbed from the atmosphere); it was present only in the first cycle and absent in the following ones. This enabled us to determine the glass transition temperature of the PUFs, which was within 92.5–95.5°C. This again corresponds to the properties of rigid PUFs.

Wastes from polyurethanes are relatively resistant to degradation, which creates an environmental risk. Therefore, we must increase the pace of degradation in order to improve this risk. We hypothesize that the application of PVA as a substrate to obtain polyols and then polyurethane foams offers an opportunity to obtain biodegradable materials.

The biodegradation of PVA-derived PUFs was tested in a soil environment. Foam samples in powder form and separately as cubic samples were placed in a soil solution prepared in a standard manner, and biological oxygen demand (BOD) was observed for 28 days. The mass percentages of C, H, N, and O were determined via an elemental analysis (Table 7). Based on these values, the theoretical BOD could be calculated and compared with the experimental BOD after 28 days of testing. The degree of degradation (Dt) of the polyol and PUFs was thus obtained (Table 8).

The degradation tests showed that a cube of PUF obtained from PVA is partially biodegradable: after 1 month of exposure to soil conditions, 54.6% had biodegraded. The powdered foam, on the other hand, was completely biodegradable under these conditions. This result is promising and indicates the possibility of obtaining rigid, biodegradable polyurethane foams based on hydroxyalkylated PVA.

## 4. Summary and Conclusions

1. A new method for the synthesis of polyol from PVA, which is suitable for obtaining rigid polyurethane foams, was elaborated.
2. The density, viscosity, and surface tension of the obtained polyol were determined. The average chemical structure of the polyol was postulated together with the well-defined admixture of low-molecular-weight products of the oxyalkylation of glycidol with ethylene carbonate and oligomers of glycidol.
3. The foams obtained from PVA-derived polyol exhibited properties typical of rigid polyurethane foams, but their thermal resistance was enhanced. The obtained PUFs can thus be exploited for a long time at a temperature of 150 °C.
4. The obtained PUFs are ecologically friendly. Powdered foam degrades completely, while a cube of PUF is degraded by up to 54.6% following 28 days of exposure to soil, based upon biological oxygen demand measurements.

## Figures and Tables

**Table 1:** Interpretation of MALDI–ToF spectrum of polyols.

Entry	Signal Position *m*/*z*	Relative Intensity of Signal [%]	Molecular Ion Structure	Calc. Molecular Weight [g/mol]
1	113.133	8.79	GL + K^+^	113.000
2	196.994	15.93	H_2_O + 2GL + CH_3_OH	198.110
3	212.897	20.61	H_2_O + 2GL + OE + H^+^	211.118
4	236.921	5.39	2GL + 2OE + H^+^	237.134
5	274.928	9.78	2GL + 2OE + K^+^	275.090
6	291.107	3.56	H_2_O + GL + 4OE + Na^+^	291.142
7	307.085	8.63	H_2_O + 3GL + OE + Na^+^	307.137
8	335.134	26.05	3GL + 2OE + Na^+^	333.153
9	351.096	80.39	H_2_O + 3GL + 2OE + Na^+^	351.163
10	363.097	19.87	4GL + OE + Na^+^	363.163
11	379.163	23.67	4G +OE + K^+^	379.137
12	393.928	89.51	2GL + 6OE-H_2_O	394.220
13	395.126	90.69	4 GL + OE + CH_3_OH + Na^+^	395.189
14	407.130	18.62	2GL + 5OE + K^+^	407.168
15	409.153	16.40	4GL + 3OE-H_2_O + H^+^	411.223
16	421.143	10.75	GL + 7OE + K^+^	421.184
17	423.165	10.30	4GL + 2OE + K^+^	423.520
18	425.119	18.54	5 GL + CH_3_OH + Na^+^	425.200
19	437.121	20.19	3GL + 4OE + K^+^	437.179
20	439.144	40.74	4 GL + 2OE + CH_3_OH + Na^+^	439.179
21	451.165	5.36	2GL + 6OE + K^+^	451.195
22	465.158	5.65	GL + 8OE + K	465.598
23	469.147	17.00	5 GL + OE + CH_3_OH + Na^+^	469.226
24	481.149	11.43	3GL + 5OE + K^+^	481.205
25	495.170	3.40	2GL + 7OE + K^+^	495.221
26	497.197	4.58	6 GL + CH_3_OH + Na^+^	499.237
27	590.901	100	H_2_O + 6GL + 2OE + K^+^ 7GL + 2OE-H_2_O + H^+^	589.247589.307

Description of abbreviations used in the table: GL—glycidol; OE—oxyethylene group; Na^+^—sodium cation; K^+^—potassium cation; -H_2_O means the dehydration of the product.

**Table 2:** The influence of the composition on the foaming process.

Composition [g/100 g of Polyol]				Isocyanate Index	Foaming Process			Product Characterization
pMDI	Water	Silicon L-6900	TEA		Cream Time [s]	Rise Time [s]	Tack-Free Time [s]	
100	2	2.9	2.0	0.8	10	5	10	Low foaming of foam
115	2	2.9	2.0	1.0	10	6	8	Viscous surface of foam
125	2	2.9	2.0	1.1	10	7	Immediate	Rigid foam, irregular pores
135	2	2.9	2.0	1.2	13	8	5	Rigid foam, irregular pores
125	2	2.9	1.1	1.1	20	10	Immediate	Rigid foam, regular pores
125	2	2.9	0.9	1.1	21	12	Immediate	Rigid foam, regular pores
125	2	2,9	0.8	1.1	25	12	Immediate	Rigid foam, regular pores
125	2	2.9	0.7	1.1	25	12	1	Rigid foam, regular pores
**125**	**2**	**2.9**	**0.4**	**1.1**	**30**	**15**	**2**	**Rigid foam, regular pores**
120	3	2.9	2.0	0.9	8	7	Immediate	Viscous surface of foam
135	3	2.9	2.0	1.1	10	8	Immediate	Rigid foam, regular pores
145	3	2.9	2.0	1.2	12	10	2	Rigid foam, irregular pores
170	3	2.9	2.0	1.4	13	11	6	Viscous surface of foam
135	3	2.9	1.0	1.1	20	5	Immediate	Rigid foam, regular pores
135	3	2.9	0.4	1.1	25	25	1	Rigid foam, regular pores
**135**	**3**	**2.9**	**0.3**	**1.1**	**30**	**28**	**1**	**Rigid foam, regular pores**
135	3	2.9	0.1	1.1	35	35	1	Semi-rigid foam

Compositions used to obtain PUFs are in bold.

**Table 3:** Some properties of foams.

Amount of Water/100 g of Polyol	Density [kg/m^3^]	Absorption of Water [wt%]			Heat Conductance Coefficient [W/m·K]	Mass Loss [wt%] After One Month Exposure at Temperature		Compressive Strength [MPa]		
		5 min	3 h	24 h		150 °C	175 °C	Before Exposure	After Exposure at Temperature	
									150 °C	175 °C
2	76.0	0.36	1.09	1.99	0.0328 ± 0.0006	15.75	35.85	0.361	0.431	0.510
3	56.1	0.50	1.59	2.15	0.0388 ± 0.0009	18.62	38.72	0.259	0.330	0.554

The density was determined with 2.1% accuracy. The standard deviation in the case of absorption of water was 1.5%. Compressive strength was determined with 2.5% accuracy.

**Table 4:** Dimensional stability [%] of foams at a temperature of 150 °C.

Amount of Water/100 g of Polyol	Length Change		Width Change		Height Change	
	20 h	40 h	20 h	40 h	20 h	40 h
2%	−1.44	−2.05	−1.36	−1.86	−1.24	−1.80
3%	−2.34	−3.52	−3.23	−3.35	−1.98	−2.92

The dimensional stability did not exceed 1.2%.

**Table 5:** Results of micrograph analysis.

Amount of Water/100 g of Polyol	Larger Diameter [µm]	Smaller Diameter [µm]	Thickness of Cell Wall
2	290 ± 38	228 ± 34	18 ± 3
3	197 ± 47	169 ± 23	9 ± 2

**Table 6:** Thermal analysis of foams determined by dynamic analysis.

Amount of Water/100 g of Polyol	T_5%_ [°C]	T_10%_ [°C]	T_25%_ [°C]	T_50%_ [°C]	T_max_ of Decomposition [°C]	Tg
2	197	235	280	346	320	95.5
3	190	228	277	338	310	92.5

T_X%_—temperature, at which x% mass loss occurs.

**Table 7:** Mass shares of individual elements in PUFs.

Element			
C	H	O	N
0.6236	0.0628	0.2484	0.2484

**Table 8:** Results of susceptibility to biodegradation of polyol and foam.

Sample	BOD_X_	BOD_28_	Sample Mass [g]	TOD Counts	TOD [mg/dm^3^]	Dt [%]
Foam—cube	87.4	28.7	0.20	10.5216	52.61	54.6
Foam—powder	1590	100.3	0.20	10.5216	52.61	100.0

## Data Availability

The original contributions presented in this study are included in the article. Further inquiries can be directed to the corresponding author.
